# Symmetry systems on the wings of *Dichromodes* Guenée (Lepidoptera: Geometridae) are unconstrained by venation

**DOI:** 10.7717/peerj.8263

**Published:** 2020-01-02

**Authors:** Sandra R. Schachat

**Affiliations:** Department of Geological Sciences, Stanford University, Stanford, CA, United States of America

**Keywords:** Color pattern, Evolution, Macroheterocera, Scale, Venation

## Abstract

The nymphalid groundplan, an idealized schematic illustrating the essential elements of butterfly wing patterns, predicts a consistent relationship between color pattern and wing venation. Moths in the family Geometridae have wing shapes and patterns that often resemble those of butterflies, and until recently, this family was believed to be among butterflies’ closest relatives. However, an examination of the geometrid genus *Dichromodes* Guenée, 1858 shows no consistent relationship between the central symmetry system and wing venation. Whereas the distal edge of the central symmetry system is predicted to reach the costal margin proximal to the Subcostal vein in butterflies and acronictine moths, it has no consistent relationship with the Subcostal, Radius, or Radial Sector 1 veins in *Dichromodes*. This finding highlights developmental diversity that was previously overlooked due to the overwhelming preference for butterflies in studies of lepidopteran wing patterns.

## Introduction

**Figure 1:**
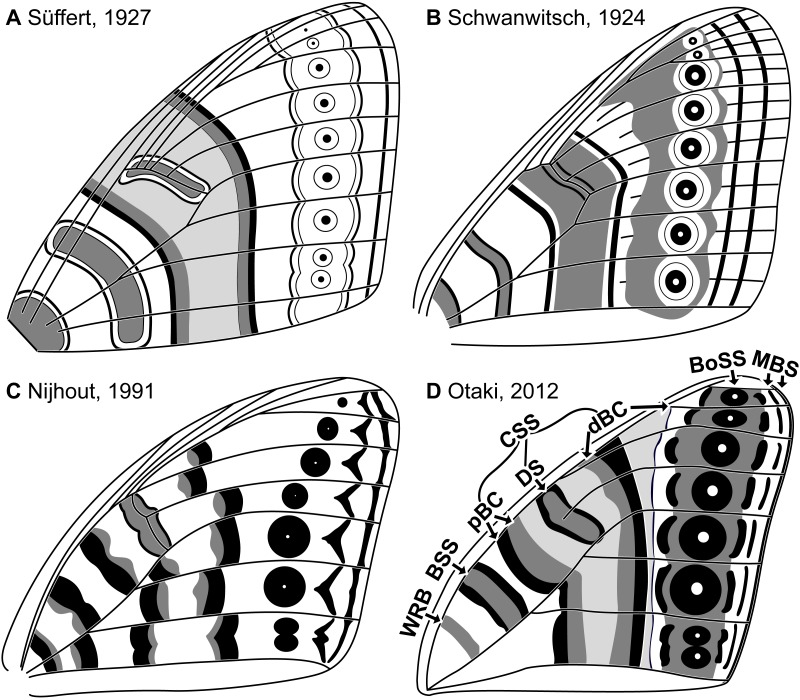
The four main versions of the nymphalid groundplan (A–D). The most recent version (D) also includes modifications based on molecular data (Martin & Reed, 2010). The elements of the nymphalid groundplan are labeled in (D), with abbreviations as follows. WRB, wing root band; BSS, basal symmetry system; CSS, central symmetry system; pBC, proximal band of the central symmetry system; DS, discal spot; dBC, distal band of the central symmetry system; BoSS, border symmetry system; MBS, marginal band system. Image credit: Süffert (1927); Schwanwitsch (1924); Nijhout (1991); Otaki (2012), modified by Sandra R. Schachat.

Throughout the twentieth century, moths in the family Geometridae were believed to be among butterflies’ closest relatives (Nijhout, 1994). The wings of geometrids and butterflies are similar in size, shape, and color pattern. Once articulated, the nymphalid groundplan (NGP)—the idealized model that describes the fundamental elements of butterfly wing patterns (Schwanwitsch, 1924; Süffert, 1927)—was swiftly put to use for the study of geometrid wing patterns (Henke, 1928; Süffert, 1929). As a result, geometrid wing patterns were described almost exclusively in terms of the presence or absence of characters initially observed in butterflies and were examined in comparatively little detail. For example, the relationship between wing venation and wing pattern was explicitly illustrated in early studies of the NGP (Fig. 1), but in the studies of geometrid wing patterns that soon followed, venation was not illustrated or discussed (Henke, 1928; Süffert, 1929; Henke, 1933; Schwanwitsch, 1956).

The focus on the wing patterns of butterflies has become more pronounced in recent decades. Modern technologies for the study of development and genetics allow for extremely detailed studies but often require colonies of animals that can be kept in a laboratory, and butterfly genera such as *Bicyclus* Kirby, 1871 (Robertson & Monteiro, 2005), *Junonia* Hübner [1819] (Kodandaramaiah, 2009), *Heliconius* Kluk, 1802 (Reed & Gilbert, 2004), *Papilio* Linnaeus, 1758 (Kunte et al., 2014), *Pieris* Schrank 1801 (Takayama & Yoshida, 1997), and *Vanessa* Fabricius 1807 (Abbasi & Marcus, 2015) are disproportionately represented among the Lepidoptera that have been successfully reared in a laboratory setting. Furthermore, traditional morphological (Otaki, 2012; Penz & Mohammadi, 2013; Taira et al., 2015) and physiological (Kusaba & Otaki, 2009; Nijhout, 2010) approaches also typically focus on butterflies alone. The popularity of moths in the family Saturniidae has increased in recent years for studies that focus on wing pattern along the termen or within the interior of the wing (Marcus, 2019; Monteiro et al., 2006; Sourakov, 2017).

However, studies of wing pattern in higher moths such as Geometridae cannot rest on the assumption that their wing patterns are simpler variants of butterfly wing patterns. There are three main reasons why this is the case. First, recent molecular phylogenetic studies have shown that geometrids are not nearly as closely related to butterflies as previously believed, and in fact belong to the more recently-derived clade Macroheterocera (Regier et al., 2009; Mutanen, Wahlberg & Kaila, 2010; Regier et al., 2013; Kawahara & Breinholt, 2014; Heikkilä et al., 2015). Second, recent studies of Macroheterocera in the noctuid subfamily Acronictinae and in the geometrid genus *Hydriomena* Hübner, [1825] found that wing pattern in these moths has a fundamentally different relationship with venation than that seen in butterflies (Schachat & Goldstein, 2018; Schachat, 2019), highlighting the possibility that morphological generalizations from butterflies may be of little relevance to Macroheterocera. Third, because butterflies are typically diurnal and other moths typically are not, their wing patterns are viewed in different circumstances, leading to different possible functions for wing pattern and selective pressures. Butterfly wing patterns are often studied in the context of intra- and interspecific signaling whereas moth wing patterns are often interpreted as providing camouflage via crypsis or disruptive coloration (Stevens & Cuthill, 2006).

The NGP consists primarily of three “symmetry systems”: concentric transverse bands of color that are symmetrical, not necessarily in shape but in color, along the proximo-distal axis of the wing (Fig. 1). As in butterflies, three symmetry systems have been observed on the wings of both Geometridae and Noctuidae; only one symmetry system has been observed on the wings of Pyralidae and Saturniidae (Nijhout, 2003). A number of recent studies have focused on wing pattern in Geometridae, in the context of ecology (Heidrich et al., 2018), developmental genetics (van’t Hof et al., 2015; Nadeau et al., 2016), and phylogeny (Strutzenberger et al., 2010; Choi, 2001). One recent contribution used the NGP as a framework for the analysis of macroheteroceran wing pattern (Martin & Reed, 2010) but included only one geometrid species and did not interrogate the importance of wing venation.

### The potential importance of wing venation

**Figure 2:**
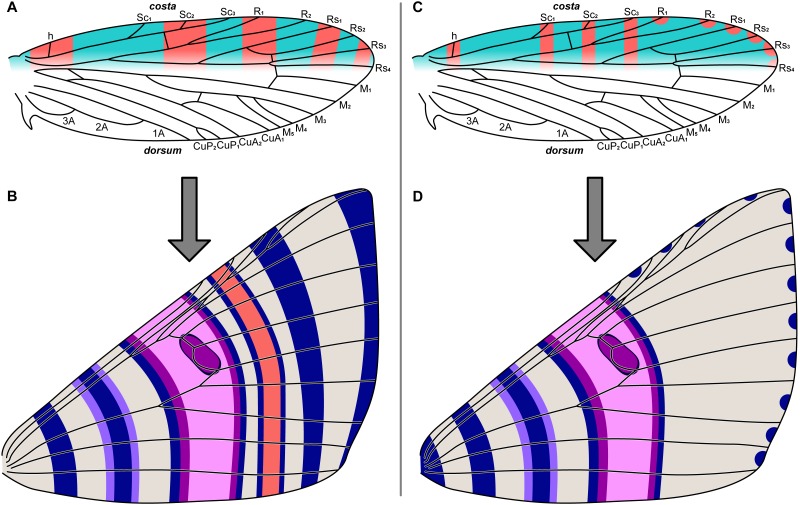
The two predictive models for wing pattern in microlepidoptera, with hypothesized origins of the nymphalid groundplan plotted onto a typical wing for the genus *Dichromodes* (based on wing slide ANIC W236). (A) The alternating wing-margin model, in which pattern elements of alternating colors straddle each vein along the costa. (B) The latest version of the NGP, which follows the constraints of the alternating wing-margin model and is consistent with observations of Nymphalidae. (C) The uniform wing-margin model, in which pattern elements of the same color straddle each vein along the costa and termen. (D) A hypothesized version of the NGP, which follows the constraints of the uniform wing-margin model and is consistent with observations of Noctuidae. Image credit: Sandra R. Schachat.

This is the second in a series of three studies examining whether the relationship between wing pattern and venation in Geometridae is consistent with the NGP (Figs. 1 and 2B), with previous observations of Macroheterocera in the family Noctuidae (Fig. 2D), or with neither. The first study in this series focused on very simple wing patterns that consist of dark and light bands (Schachat, 2019). The present study focuses on wing patterns that appear to conform to the nymphalid groundplan in that they contain at least one symmetry system. The last study in this series will focus on “higher-level” symmetry systems, which occur in geometrids but not in butterflies (Süffert, 1929).

The relationship between wing pattern and venation has been classified with two models originally based on microlepidoptera. The first, the “alternating wing-margin model” (Baixeras, 2002; Brown & Powell, 1991; Schachat & Brown, 2015; Schachat & Brown, 2016), predicts that alternating light and dark bands of color straddle each wing vein along the costa (Fig. 2A). All available evidence indicates that this model represents the ancestral state for Lepidoptera (Schachat & Brown, 2016). The second, the “uniform wing-margin model” (Schachat & Brown, 2016; Schachat & Brown, 2018; Schachat, 2017b; Schachat, 2017a), predicts that pattern elements of the same color straddle every wing vein along the costa (Fig. 2C). These models are a focus of the present contribution because the former was validated when it was used to predict the existence and the precise location of an ancestral wing vein in moths (Schachat & Gibbs, 2016) and the latter holds in nearly all lepidopteran lineages examined for which the former does not (Schachat, 2017a).

Both of these models are relevant to the NGP. In nymphalid butterflies the NGP conforms to the alternating wing-margin model (Schachat & Brown, 2016), and in acronictine noctuids the NGP conforms to the uniform wing-margin model (Schachat & Goldstein, 2018). The NGP can be plotted onto a hypothetical geometrid wing according to either of these models, providing testable hypotheses.

If the NGP develops in geometrids according to the predictions of the alternating wing-margin model (Fig. 2A), as it does in nymphalid butterflies, then pattern elements located distal to the central symmetry system (CSS) will straddle alternating veins along the costa (Fig. 2B). If the NGP develops in geometrids according to the predictions of the uniform wing-margin model (Fig. 2C), as it does in acronictine moths, then pattern elements located distal to the CSS will occur on or between all adjacent veins that terminate along the costa and termen (Fig. 2D).

The influence of ancestral wing venation provides an additional opportunity to distinguish between the two models. Wing patterns that develop according to the alternating wing-margin model are strongly influenced by ancestral lepidopteran veins that may not be present in the adult wing (Baixeras, 2002; Brown & Powell, 1991; Schachat & Brown, 2015; Schachat & Brown, 2016; Schachat & Gibbs, 2016). However, this constraint does not appear to hold for wing patterns that develop according to the uniform wing-margin model: in areas where ancestral veins are not present on the adult wing, it is possible to observe more (Schachat, 2017b) or fewer (Schachat & Brown, 2016; Schachat, 2017a; Schachat & Brown, 2018) pattern elements than would be expected based on the configuration of those veins. Of note, the predictions of these two hypotheses overlap somewhat: each predicts that the distal edge of the central symmetry system reaches the costa proximal to the distalmost branch of the Subcostal vein (Figs. 1, 2B and 2D), a prediction that dates to the inception of the NGP (Schwanwitsch, 1924; Süffert, 1927) and is supported by recent studies (Nijhout, 1991; Otaki, 2012; Penz & Mohammadi, 2013).

**Figure 3:**
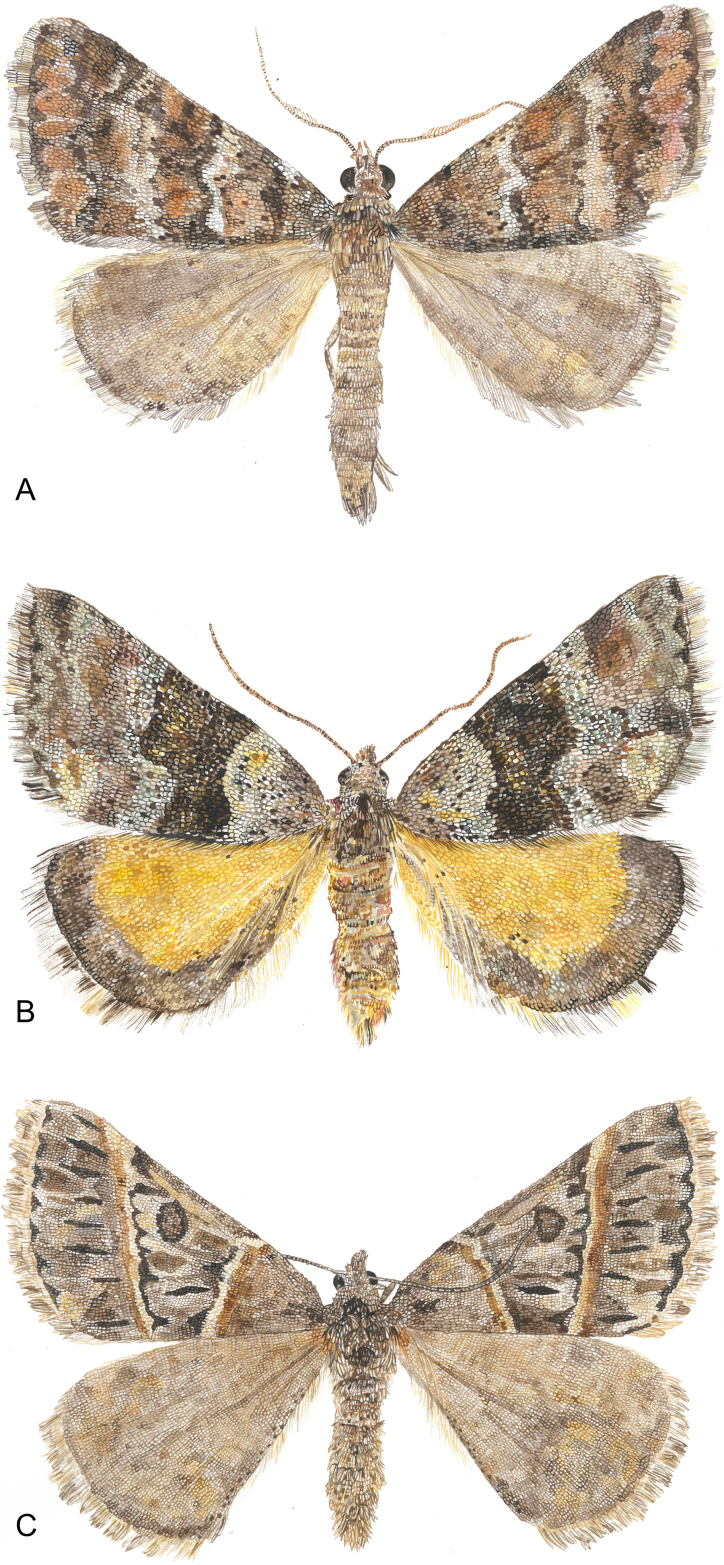
Watercolor illustrations of three *Dichromodes* species. (A) *D. fulvida*. (B) *D. ainaria*. (C) *D. orthotis*. Image credit: Celia L. Curtis.

The genus *Dichromodes* Guenée, 1858 (Scoble, 1999) was chosen for this study because it contains many species with easily identifiable central symmetry systems, but does not contain the higher-level symmetry systems observed in other geometrids (Süffert, 1929), thus facilitating comparisons with butterfly wing patterns as epitomized by the nymphalid groundplan (Fig. 3). Because geometrids in the subfamily Larentiinae are vastly overrepresented in morphological studies of wing pattern (Henke, 1928; Süffert, 1929; Henke, 1933; Schwanwitsch, 1956; Schachat, 2019), *Dichromodes* expands the phylogenetic scope of this body of knowledge. The primary aim of this contribution is to determine whether the most prominent symmetry system on the wings of *Dichromodes* conforms to the alternating wing-margin model, the uniform wing-margin model, or neither model. The wing patterns of *Dichromodes* are also compared to a historical classification scheme based primarily on moths in its taxonomic family, Geometridae.

## Methods

All specimens examined are held in the Australian National Insect Collection (ANIC) in Canberra, Australia. Species from both Australia and New Zealand have been assigned to *Dichromodes*. The species that were initially assigned to this genus occur in Australia, where the larvae feed on the leaves of Myrtaceae (Common, 1990); the putative *Dichromodes* species from New Zealand have larvae that feed on lichens, leading some authors to question whether these species should be assigned to a separate genus (Hoare, Millar & Richardson, 2016). The present study focuses on *Dichromodes* species occurring in Australia, where a total of 67 described species of *Dichromodes* occur. *Dichromodes* was originally assigned to the subfamily Oenochrominae (Nielsen, Edwards & Rangsi, 1996), which was found to be polyphyletic (Sihvonen et al., 2011). The only molecular phylogenetic study to include *Dichromodes* was published recently, and assigned this genus to the newly-erected geometrid subfamily Epidesmiinae Murillo-Ramos, Sihvonen & Brehm (Murillo-Ramos et al., 2019). This new subfamily corresponds to the “slender-bodied” lineage that Holloway (1996) removed from Oenochrominae *s. str*.

Because the wings of Geometridae are densely scaled, the clearing agent Histolene was applied to render wing venation visible; this is a semi-destructive method because the clearing agent leaves a permanent residue on *Dichromodes*’ wings. For this reason, each sex of each species was examined only if five or more specimens were available. Species were excluded from the study if their wing patterns have only one color. Only forewings were examined because the hindwings do not contain any discrete pattern elements. The wing slide ANIC W236 was used as a reference for wing venation.

### Illustrations

One specimen was examined per sex/species. The more intact of the two forewings was chosen; only one forewing could be examined per specimen because the application of Histolene made the entire specimen greasy, thus rendering the other forewing unusable. All photographs were taken with a Leica DFC500 camera through a Leica M205C microscope, with Leica Application Suite software version 4.4. First, the forewing was photographed in its original condition, without the clearing agent. Then, without moving the specimen, the clearing agent was applied, the specimen was illuminated from below to help see the veins, and the forewing was photographed again. A vector drawing of the wing venation was created in Affinity Designer graphics software version 1.5.5 by tracing the veins from the second photograph. The vector drawing of the veins was then superimposed on the first photograph to visualize the natural wing pattern and venation simultaneously.

Because fringe extends beyond the wing margin in *Dichromodes*, the wing veins do not reach the apparent edge of the wing as seen in photographs. Dashed lines were used for all inferred veins that could not be observed in the specimen. Color coding was used for the veins that reach the costa: Sc is in bright red, R is in cyan, Rs_1_ is in purple, Rs_2_ is in dark blue, and Rs_3_ is in pale red. In all illustrations, an arrow indicates the distal edge of the CSS along the costa.

### Terminology and classification

“Costa” refers to the costal, or anterior, margin of the wing; “termen” to the distal margin of the wing; and “posterior margin” to the anal margin of the wing, which is referred to by some authors as the “dorsum” (Carter & Kristensen, 1998). The terminology used for venation follows conventions that are widely accepted in studies of all winged insect orders (Wootton, 1979). Although the Radius and Radial sector veins are often conflated as branches of “R” in the Macroheterocera literature, Wootton’s terminology, which has been used in many larger-scale studies of Lepidoptera (Carter & Kristensen, 1998), is used here. According to this terminological scheme, geometrids have an unbranched “R” vein followed by four branches of the “Rs” vein: Rs_1_, Rs_2_, Rs_3_, and Rs_4_ (Fig. 2).

In the NGP, “background color” is the color that surrounds darker pattern elements such as parafocal elements and symmetry systems. Recent studies have found that color is not a reliable indicator of homology, and that dark and light colors can “flip” between pattern elements (Schachat & Brown, 2018); this is observable even in the Micropterigidae, a mandibulate family that includes some species with NGP-like wing patterns (Schachat & Brown, 2016). The term “ground color,” typically used in taxonomic descriptions of moth wing patterns, can therefore be misleading in the context of homology. Here, the term “background” is used simply because of its inclusion as a component of the NGP (Otaki, 2012).

The most salient feature of the NGP is the central symmetry system (CSS). The terminology used here for the CSS and other elements of the NGP comes from Otaki (2012) and is illustrated in Fig. 1. The CSS surrounds the discal spot (DS) and is bounded by the proximal band of the central symmetry system (pBC) and the distal band of the central symmetry system (dBC).

In addition to the background color, various elements of the NGP occur proximal to the CSS. Most proximal, at the base on the wing, is the wing root band (WRB), consisting of a single color. Between the WRB and the CSS is the basal symmetry system (BSS). The bands within the BSS develop so that they are self-symmetrical in color, as is also the case for the CSS, but the BSS more often contains only two colors, and lacks additional features such as the discal spot.

Two additional elements of the NGP occur distal to the CSS. Closest to the CSS is the border symmetry system (BoSS), which often develops into eyespots in nymphalids. Most distal is the marginal band system (MBS), typically in a single color. The MBS includes the pattern elements that reach the termen. Spots that reach the termen on butterfly wings, between adjacent veins, are called “parafocal elements” (Nijhout, 1990). Here, this term is used for pattern elements that resemble the parafocal elements identified by Nijhout (1990) on the wings of Nymphalidae; as is the case with all other elements of the NGP, such as the symmetry systems, this terminology is used in a strictly descriptive sense and is not intended to imply homology with butterflies or any other lineage of Lepidoptera. Parafocal elements on the wings of Macroheterocera (Schachat & Goldstein, 2018) often resemble the parafocal elements 86, 87, 88, 89, 97, and 98 in Nijhout’s classification system developed for butterflies (Nijhout, 1990).

**Figure 4:**
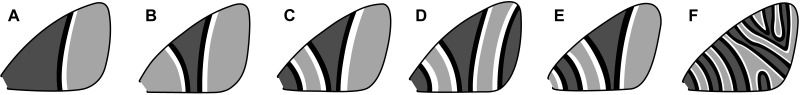
Henke’s categorization scheme for symmetry system complexity, with the categories arranged from least (A) to most (F) complex. All categories are based on observations of individual moth species, as follows. (A) *Lasiocampa quercus* Linnaeus, 1758 (Lasiocampidae). (B) *Campaea margaritaria* Linnaeus, 1761 (Geometridae). (C) *Thera juniperata* Linnaeus, 1758 (Geometridae). (D) *Eulithis testata* Linnaeus, 1761 (Geometridae). (E) *Perizoma sagittata* Fabricius, 1787 (Geometridae). (F) *Syricoris rivulana* Scopoli, 1763 (Tortricidae). Image credit: Henke (1933), vectorized by Sandra R. Schachat.

An early classification scheme, based primarily on Geometridae, categorizes symmetry systems according to their complexity (Henke, 1933). This scheme includes six categories (Fig. 4) ranging from a single, incomplete symmetry system (Fig. 4A) to multiple symmetry systems that may become confluent in the interior of the wing (Fig. 4F). In the first category (Fig. 4A), the entire proximal portion of the wing is covered by a pattern element that resembles the distal half of a symmetry system—with no discal spot or any other features—and the entire distal portion of the wing has nothing but the background color. The second category (Fig. 4B) contains a single, highly rudimentary symmetry system. The third category (Fig. 4C) contains a complete CSS, plus a BSS that extends to the base of the wing with its proximal portion missing. The fourth category (Fig. 4D) contains the features of the third, plus a BoSS that extends to the termen with its distal portion missing. The fifth category (Fig. 4E) contains a complete BSS and CSS, with no BoSS. The sixth and final category (Fig. 4F) contains eight symmetry systems, some of which become confluent and do not extend entirely from the costa to the posterior margin. The wing patterns of *Dichromodes* were evaluated according to this scheme.

### Identification of the central symmetry system

Two criteria were used to identify the CSS, both consistent with Schwanwitsch’s identification of the CSS in the geometrid *Eulithis destinata* Packard, 1873 in that the CSS is a transverse pattern element that reaches the costa and encompasses the discal spot (Schwanwitsch, 1956).

The first criterion identifies the CSS as the pattern element that surrounds the discal spot and whose distal edge is adjacent to a line or color field in the lightest shade on the wing. This criterion is taken from the description of the CSS for butterflies, according to which the proximal and distal bands at either edge of the CSS are the darkest, the color field between these two bands—comprising the interior of the CSS—is of an intermediate shade, and the wing surface surrounding the CSS is of a lighter shade (Nijhout, 1991; Otaki, 2017). This definition of the CSS has been used for Geometridae beginning with the first extensive application of the NGP to this family (Henke, 1928), in which the CSS was called the *Zentralkern* and was defined as the area bounded by the two *Zentralbinden*, which represent the darkest color on the wing. These *Zentralbinden* correspond to the pBC and dBC of the NGP.

The second criterion identifies the CSS as the largest possible self-symmetrical pattern element that surrounds the discal spot, does not extend to the base of the wing, and does not abut the closest pattern element that occurs distally on the wing. Accordingly, the CSS can include the lightest color on the wing even if this color does not occur in the central color field between the pBC and dBC. This criterion was used in recognition of the recent finding that color can be misleading in the context of wing pattern homology (Schachat & Brown, 2016; Schachat & Brown, 2018). This criterion is also supported by Penz & Mohammadi (2013)’s recent study of nymphalid butterflies: on the forewing of *Caligopsis seleucida* Hewitson, 1877, the lightest color on the wing occurs within the CSS, and on the forewing of *Eryphanis bubocula* Butler, 1872, the same phenomenon occurs and the lightest color on the wing does not even border the CSS distally.

The second criterion is less straightforward because of the frequent difficulty in differentiating the adjacent, distal pattern element from the background color. Therefore, the photographs, Table 1, and the Kolmogorov–Smirnov test are all organized according to the first criterion.

**Table 1:** The species examined here, taxonomic authorities, the point where the distal edge of the CSS reaches the costa, and figure references. “B/t” is an abbreviation for “between.”

**Species**	**Authority**	**Distal edge of CSS at costa**	**Figure(s)**
*D. ainaria*	Guenée, 1858	B/t Sc & R	Figures S2A (f), S2B (m)
*D. anelictis*	Meyrick, 1890	Sc	Figures S1A (f), S1B (m)
*D. aristadelpha*	Lower, 1903	Sc	Figures S1C (m)
*D. atrosignata*	Walker, 1861	B/t R & Rs_1_ (f), at Rs_1_ (m)	Figures S6A (f) Fig. 5F (m)
*D. compostis*	Meyrick, 1890	B/t Sc & R	Figures S2C (f), S2D (m)
*D. confluaria*	Guenée, 1857	Rs_1_	Figure S6B (m)
*D. denticulata*	Turner, 1930	Sc	Figure S1D (m)
*D. estigmaria*	Walker, 1861	R	Figures S5A (f), S5B (m)
*D. euprepes*	Prout, 1910	B/t Sc & R	Figures S3A (f), S3B (m)
*D. explanata*	Walker, 1861	B/t Sc & R (f), at R (m)	Figures S2E (f), S5C (m)
*D. exsignata*	Walker, 1861	B/t Sc & R	Figure S2F (f)
*D. indicataria*	Walker, 1866	B/t R & Rs_1_ (f), b/t Sc & R (m)	Figures 5E (f), S3C (m)
*D. leptozona*	Turner, 1930	Rs_1_	Figures S6C (f), S6D (m)
*D. mesodonta*	Turner, 1930	B/t Sc & R	Figure 5C (f)
*D. mesoporphyra*	Turner, 1939	Sc	Figure S1E (m)
*D. mesotoma*	Turner, 1943	R	Figure S5D (f)
*D. molybdaria*	Guenée, 1858	R	Figures S5E (f), S5F (m)
*D. ornata*	Walker, 1861	Rs_1_	Figures S7A (f), S7B (m)
*D. orthotis*	Meyrick, 1890	B/t Sc & R	Figure S3D (m)
*D. orthozona*	Lower, 1903	R	Figure 5D (f)
*D. paratacta*	Meyrick, 1890	Sc	Figure 5B (m)
*D. rimosa*	Prout, 1910	B/t Sc & R	Figure S4A (m)
*D. rufilinea*	Turner, 1939	B/t Sc & R	Figure S4B (f)
*D. rufula*	Prout, 1910	Sc	Figure S1F (m)
*D. scothima*	Prout, 1910	B/t Sc & R	Figure S4C (f)
*D. semicanescens*	Prout, 1913	Proximal to Sc?	Figure 5A (m)
*D. stilbiata*	Guenée, 1858	Rs_1_	Figures S7C (f), S7D (m)
*D. triparata*	Walker, 1861	B/t Sc & R	Figure S4D (f)

In each photograph, an arrow indicates the point where the distal edge of the CSS reaches the costa. If the distal edge of the CSS varies according to the criterion used, two arrows are used. The proximal edge of the CSS is not indicated in the figures because it cannot be used to evaluate concordance with either predictive model.

### Kolmogorov–Smirnov tests

The points at which the distal edge of the CSS reaches the costa in different specimens form a discrete, ordered distribution. This distribution was visualized with a histogram produced in the R package ggplot2 version 2.2.1 (Wickham, 2009). Kolmogorov–Smirnov tests were used to test whether this distribution is consistent with either a binomial or a uniform distribution.

First, a uniform and a binomial distribution were simulated in R version 3.4.2 (R Development Core Team, 2017). The binomial distribution was chosen because it offers a discrete approximation of a normal distribution and was simulated using the base-R function dbinom. Both distributions were simulated with 1,000 points. Kolmogorov–Smirnov tests were then performed, using the base-R function ks.test, to compare the observed distribution to the uniform and binomial distributions. A variant of the Kolmogorov–Smirnov test created for discrete data was also performed, using the ks.boot function in the R package Matching version 4.9-2 (Sekhon, 2011). Because the *p*-values returned from the ks.test and ks.boot functions are identical, only one *p*-value is reported for each distribution.

## Results

Forty specimens belonging to the genus *Dichromodes* were examined, representing 28 species (Table 1). According to the criteria outlined above, the proximal and distal edges of the CSS can be distinguished in all specimens examined here.

### The central symmetry system

**Figure 5:**
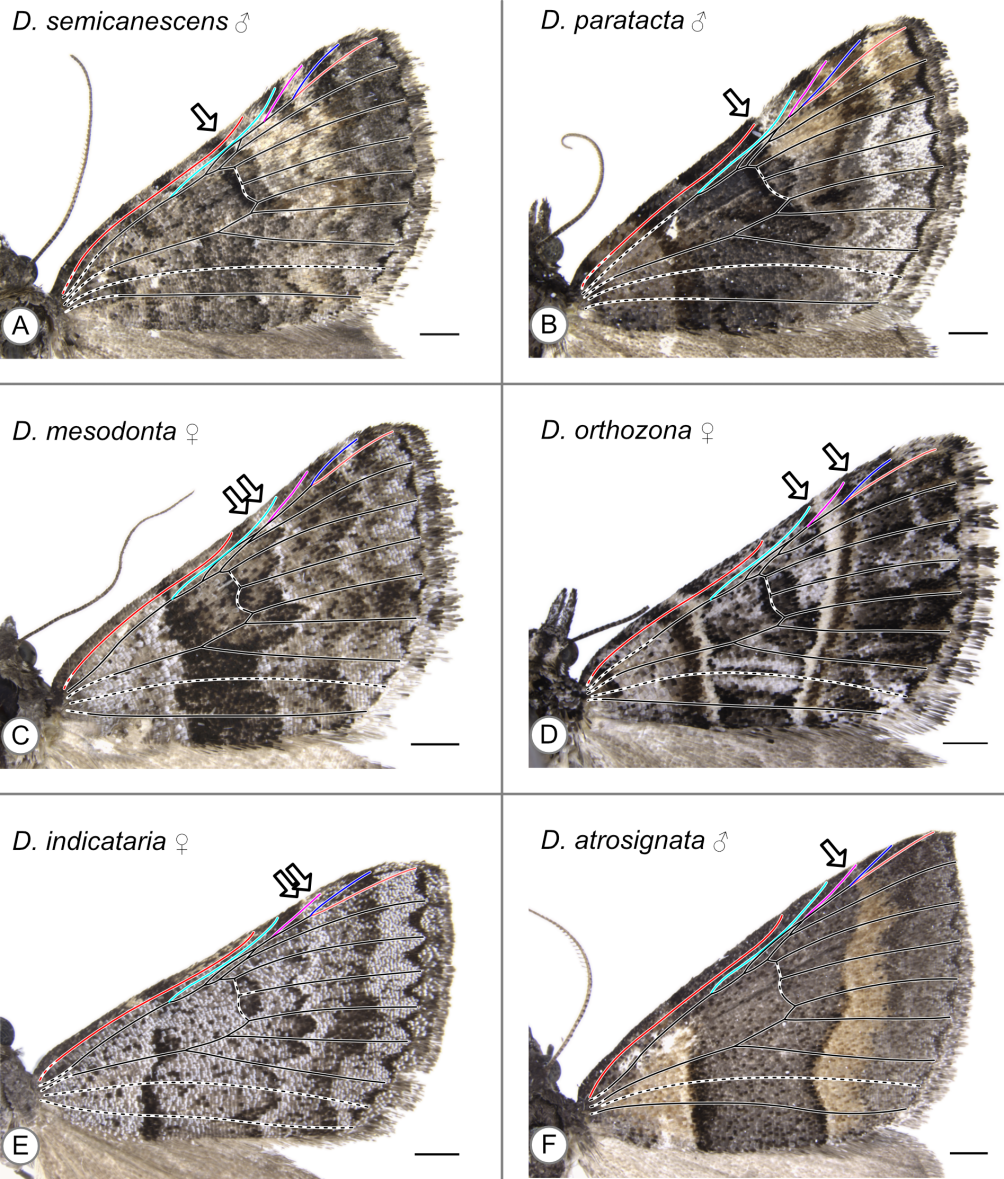
Wing patterns in the genus *Dichromodes*, with wing venation superimposed. Arrows indicate the point where the distal edge of the central symmetry system terminates along the costa. (A) *D. semicanescens*, male. (B) *D. paratacta*, male. (C) *D. mesodonta*, female. (D) *D. orthozona*, female. (E) *D. indicataria*, female. (F) *D. atrosignata*, male. Image credit: Sandra R. Schachat.

With the sole exception of *Dichromodes semicanescens* Prout, 1913 (Fig. 5A) all specimens clearly violate both hypotheses presented here—based on the alternating wing-margin model and the uniform wing-margin model (Fig. 2)—in that the distal edge of the CSS reaches the costa distal to the point where Sc terminates. In different *Dichromodes* specimens, the CSS terminates at Sc (Fig. 5B, Fig. S1), between Sc and R (Fig. 5C, Figs. S2, S3, S4), at R (Fig. 5D, Fig. S5), between R and Rs_1_ (Fig. 5E, Fig. S6A), or at Rs_1_ (Fig. 5F, Fig. S6B, S6C, S6D, S7).

Both a male and female specimen were examined for 12 of the 28 species included in this study. The location of the CSS is consistent between the sexes in nine out of the twelve, or 75%, of these species (Table 1). The three exceptions are as follows. In *Dichromodes atrosignata* Walker, 1861, the CSS reaches the costa between R and Rs_1_ on the wing of the female specimen (Fig. S6A) and at Rs_1_ on the wing of the male specimen (Fig. 5F). In *Dichromodes explanata* Walker, 1861, the CSS reaches the costa between Sc and R on the wing of the female specimen (Fig. S2E) and at R on the wing of the male specimen (Fig. S5C). In *Dichromodes indicataria* Walker, 1866, the CSS reaches the costa between R and Rs_1_ on the wing of the female specimen (Fig. 5E) and between Sc and R on the wing of the male specimen (Fig. S3C).

The color of the interior of the CSS, surrounding the discal spot, often matches the background color of the wing. Typically, the CSS is a contiguous pattern element that spans the wing in a transverse direction from the costa to the posterior margin. In the male representative of *Dichromodes anelictis* Meyrick, 1890 (Fig. S1B) the CSS consists of two separate pattern elements, one reaching the costa and one reaching the posterior margin; the female belonging to this same species has a typical, contiguous CSS (Fig. S1A).

The pBC and dBC are not always continuous along the entirety of the anterior-posterior axis of the wing but can always be identified along at least part of the margin of the CSS. In some specimens, the dBC in particular is faintest toward the costa (Fig. 5F) or cannot be distinguished at all along the costa (Fig. 5C, Fig. S6A). In other specimens, the pBC and dBC are most easily distinguished near the costa (Figs. S1E, S5A, S5B, S5F). This is the case for *Dichromodes orthotis* Meyrick, 1890 (Fig. S3D), a species whose wing pattern is unique in that the pBC and dBC also form triangular shapes straddling the veins that cross the CSS. The pBC is nearly, but not entirely, complete in the female representive of *D. indicataria* (Fig. 5E). It is difficult to discern in *Dichromodes rimosa* Prout, 1910 (Fig. S4A), *Dichromodes scothima* Prout, 1910 (Fig. S4C), the male representative of *Dichromodes estigmaria* (Fig. S5B), and in *Dichromodes ornata* Walker, 1861 (Figs. S7A, S7B).

In the specimens examined here, the discal spot is outlined in the color of the pBC and dBC; the interior of the CSS, excepting the discal spot, is the same color or is of a lighter color. The only specimen for which the discal spot cannot be distinguished is *D. scothima*, which has a CSS that is uniformly dark in color (Fig. S4C); however, the consistent relationship between venation and the discal spot allows its location to be inferred.

#### Disagreement between criteria

Regardless of the criterion used to identify the distal edge of the CSS, its location along the costa varies greatly in *Dichromodes*. In 25 of the 40 specimens examined, the two criteria yield conflicting results. In 13 of these 25 specimens, the use of the second criterion expands the “CSS” to include a light band (Figs. 5C, 5E, Figs. S1D, S2A, S2B, S3A, S3B, S3C, S4B, S5D, S6A, S6C, S6D). In 11 of these 25 specimens, the use of the second criterion expands the “CSS” to include a light and a dark band (Fig. 5D, Figs. S1A, S1B, S1C, S2C, S2D, S2E, S2F, S3D, S4D, S5C). Finally, in one of these 25 specimens, the representative of *Dichromodes confluaria* Guenée, 1857, the use of the second criterion expands the “CSS” to include one dark and two light bands (Fig. S6B).

In *D. ornata* (Figs. S7A, S7B), the only species examined here whose “CSS” is asymmetrical in color and therefore reminiscent of Henke’s simplest CSS category (Fig. 4A), the proximal half of the CSS is so lightly shaded that it is impossible to determine whether the light band that occurs at the distal edge of the CSS has a counterpart at the proximal edge of the CSS (Figs. S6A, S6B). In *Dichromodes stilbiata* Guenée, 1858, light bands occur at both the proximal and distal edges of the CSS but these narrow so sharply toward the costa that the use of the second criterion does not change the putative location of the distal edge of the CSS (Figs. S6C, S6D).

#### Quantitative analyses

**Figure 6:**
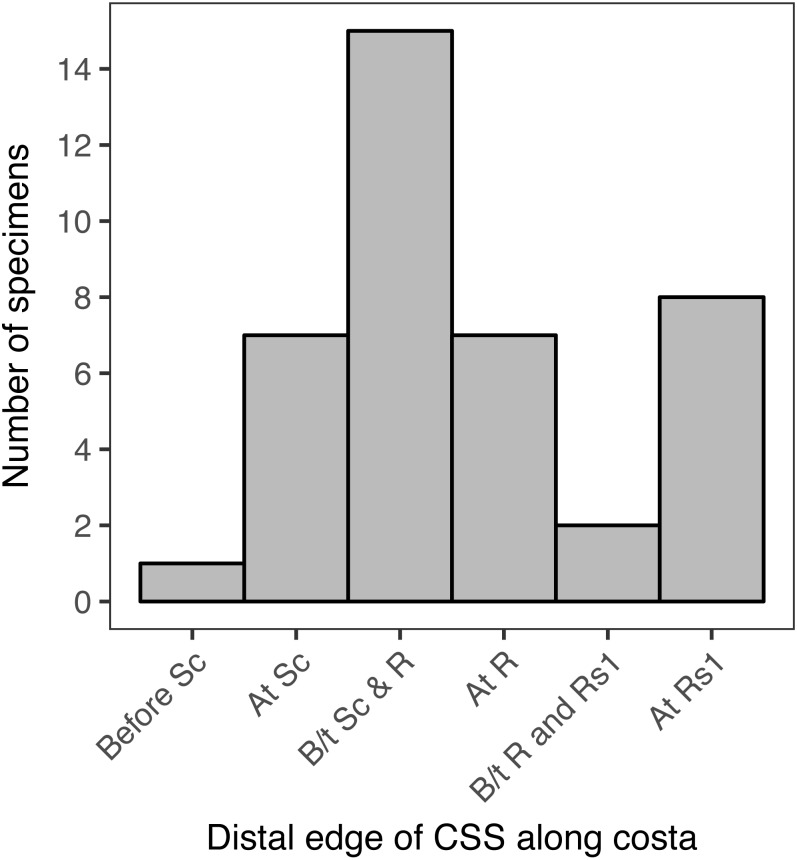
The number of specimens belonging to each of six categories, corresponding to the point where the distal edge of the central symmetry system (CSS) terminates along the costal margin of the wing. *D. semicanescens* is considered here to have a CSS whose distal edge reaches the costa before Sc, but see the text for discussion. “B/t” is an abbreviation for “between.”.

When the location of the distal edge of the CSS in each specimen is visualized with a histogram, the distribution is multimodal and does not resemble any canonical distributions such as normal, uniform, lognormal, etc. (Fig. 6). In 15 of the specimens examined here, representing 37.5% of the total, the distal edge of the CSS reaches the costa between Sc and R. In eight specimens, 20% of the total, the distal edge of the CSS reaches the costa at Rs_1_. In seven specimens, 17.5% of the total, the distal edge of the CSS reaches the costa at Sc, and in another seven specimens the distal edge of the CSS reaches the costa at R. The distal edge of the CSS reaches the costa between R and Rs_1_ in only two specimens, and it reaches the costa before Sc in only one specimen. When the “at Rs_1_” category is excluded, the distribution appears to be normal (Fig. 6). However, this category includes 20% of all specimens.

The Kolmogorov–Smirnov test confirms that the distribution of the location of the distal edge of the CSS is significantly different from a binomial distribution (*p* = 0.005**) and from a uniform distribution (*p* = 0.005**). A significant result is also recovered if *Dichromodes semicanescens* is removed from the dataset (*p* = 0.013* for both distributions).

### Other elements of the NGP

With two exceptions, the wing patterns of *Dichromodes* do not conform to any of Henke’s categories (Fig. 4). The male representative of *Dichromodes molybdaria* Guenée, 1858 (Fig. S5E) has very faint markings outside of its CSS and therefore conforms to Henke’s second category (Fig. 4B). The wing pattern of *D. atrosignata* (Fig. 5F) appears to be consistent with Henke’s fourth category (Fig. 4D). Beyond the CSS, the wing patterns of *Dichromodes* are more complex toward the termen than toward the base of the wing and so these fall outside the scope of Henke’s categories. As noted above, the CSS of *Dichromodes ornata* is asymmetrical in color and resembles Henke’s simplest category (Figs. S7A, S7B, Fig. 4A). Wing pattern in this species does not contain any elements proximal to the CSS, such as the wing root band or basal symmetry system. However, *Dichromodes ornata* does not correspond precisely to Henke’s first category because it has multiple pattern elements that occur distal to the CSS: medium-to-dark brown markings in the location of the BoSS, and dark parafocal elements.

All other species of *Dichromodes* examined here have a symmetrical CSS, but there is considerable variation in the additional elements of the NGP. The two elements of the NGP that occur proximally to the CSS—the wing root band and basal symmetry system—typically cannot be identified. This area of the wing often has mottled coloration. Many specimens lack any pattern elements in this area and simply have a few dark speckles formed by isolated dark scales (Fig. 5E, Figs. S2A, S2B, S3C, S3D, S4B, S4C, S5A, S5B, S5D, S5E, S5F, S6A, S6C, S6D, S7A, S7B). These specimens often have darker scales along the costa (Fig. 5E, Figs. S3D, S5F, S6A, S6C, S6D, S7A, S7B). A few specimens have spots at the base of the wing (Fig. 5C) or near it (Fig. 5D, Fig. S1E), and these spots can be quite faint (Fig. 5B, Fig. S1F). Some specimens have variation in scale color in this area without any discernible boundaries to permit the identification of discrete pattern elements (Figs. 5A, 5F, Figs. S1C, S2E, S2F). When distinguishable pattern elements do appear in this area they typically do not resemble the wing root band or the basal symmetry system (Figs. S1B, S2C, S2D, S3A, S3B, S4A, S4D, S5C, S6B, S7C, S7D). Only two specimens, the male representatives of *D. anelictis* and *Dichromodes denticulata* Turner, 1930, have pattern elements in this area that appear to be consistent with the NGP (Fig. S1A, S1D).

Distal to the CSS, where the border symmetry system occurs in the NGP, the *Dichromodes* specimens typically have a pattern element that is darker than the background color but is simpler than a symmetry system. This pattern element is usually of variable width (Fig. 5E), does not always reach the posterior margin (Fig. 5F), and is not always contiguous (Fig. S1E).

The parafocal elements occur along the termen only, violating the prediction from noctuid moths (Fig. 2D). Certain specimens have a pattern element between Rs_2_ and Rs_3_, near the apex of the wing, that is identical in color to the parafocal elements but is so severely flattened that it appears as a line (Figs. 5A, 5B, 5E, Figs. S1B, S1F, S2D, S3A, S3B, S3D, S5A, S5B, S5E, S6C, S6D, S7A, S7B). However, other specimens have this same pattern element in a lighter color than the parafocal elements (Figs. S1C, S1D, S1E, S4B), suggesting that this pattern element is not a parafocal element. The parafocal elements of *Dichromodes* are consistently triangular in shape, most closely resembling Nijhout’s parafocal element 86 (Nijhout, 1990). The triangular parafocal elements vary in concavity, in whether they extend to the wing veins, and in whether their distal edge lies entirely along the termen. Some specimens have parafocal elements whose shape is more ovoidal (Fig. S7B) or rectangular (Fig. S6D).

## Discussion

**Figure 7:**
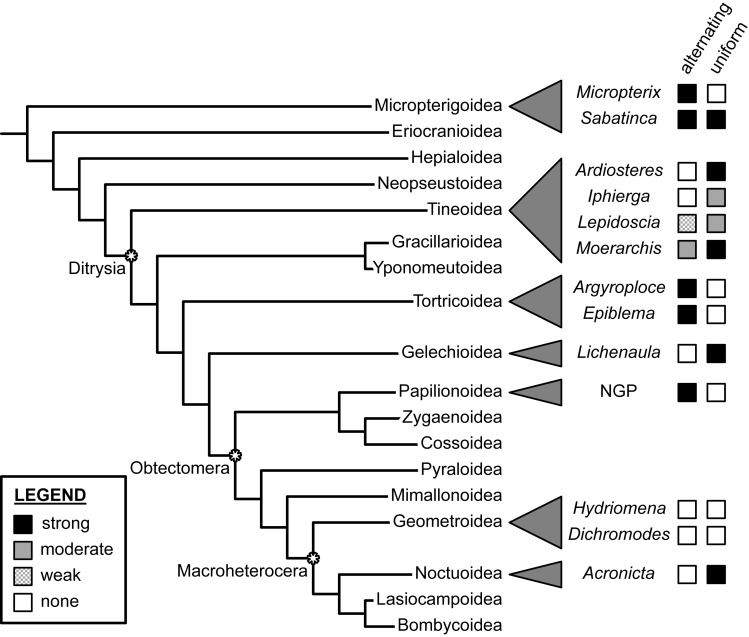
Support for the “alternating” and “uniform” wing margin models, in phylogenetic context. Geometridae, including *Dichromodes*, is the only lepidopteran family examined thus far in which there is no support for either model. Adapted from Schachat (2017a) and Schachat & Goldstein (2018). Evaluations of taxa other than *Dichromodes* in this figure are based on findings published in Schachat & Brown (2015), Schachat & Brown (2016), Schachat & Brown (2018), Schachat (2017b), Baixeras (2002), Brown & Powell (1991), Schachat (2017a), Schwanwitsch (1924), Süffert (1927), Schachat (2019) and Schachat & Goldstein (2018).

*Dichromodes* provides the first documented example of wing patterns that resemble the NGP but violate the predictions of both the alternating and uniform wing-margin models (Fig. 7). This finding is consistent with the hypothesis of separate origins of NGP-like wing patterns in Micropterigidae, Nymphalidae, Noctuidae, and Geometridae. Not only do the wing patterns of *Dichromodes* violate all predictions about the relationship between the central symmetry system and wing venation, but the highly variable nature of wing pattern within this genus also suggests that wing venation does not exert nearly as strong of a constraint in Geometridae as it does in more early-diverging lineages, such as Micropterigidae and Papilionoidea, and in other macroheteroceran lineages such as Noctuidae. This finding in *Dichromodes* is consistent with the first contribution in this three-part series on wing pattern in Geometridae, which found no relationship between wing pattern and venation along the costa in *Hydriomena* species with far simpler wing patterns than those of *Dichromodes* (Schachat, 2019).

At present, the relationship between wing pattern and venation has been investigated in only a tiny fraction of obtectomeran lineages. And yet, between the three obtectomeran superfamilies with NGP-like patterns that have been studied in recent years—Papilionoidea, Noctuoidea, and now, Geometroidea—there is as much developmental disparity as possible, in that wing patterns of Papilionoidea as described by the NGP are consistent with the alternating wing-margin model, wing patterns of the noctuid subfamily Acronictinae are consistent with the uniform wing-margin model, and wing patterns of the geometrid genus *Dichromodes* are not consistent with either.

The parafocal elements of *Dichromodes* are also different from those of Acronictinae. Most importantly for questions of homology, the parafocal elements of *Dichromodes* occur only along the termen. In contrast, parafocal elements occur along both the termen and the distal portion of the costa in many species of *Acronicta* Ochsenheimer, 1816 (Schachat & Goldstein, 2018), in an arrangement reminiscent of many microlepidopteran wing patterns (Schachat & Brown, 2016; Schachat, 2017b; Schachat, 2017a) that conform to the uniform wing-margin model (Fig. 2C). Although the relevance of the alternating and uniform wing-margin models to *Dichromodes* cannot be ascertained from the CSS because its relationship with wing venation is so variable, the finding that its parafocal elements are confined to the termen suggests that the uniform wing-margin model is of little or no relevance to the development of wing pattern in this genus. Also of note, the parafocal elements of *Dichromodes* are far less variable than those of *Acronicta* (Schachat & Goldstein, 2018) in both size and shape.

The Kolmogorov–Smirnov tests suggest that the location of the distal edge of the CSS does not follow a predictable distribution and therefore cannot be attributed to known developmental processes. The low degree of intraspecific variation suggests that the distributions used for the Kolmogorov–Smirnov tests reflect the diversity of *Dichromodes* as a whole and that the slight overrepresentation of male specimens in this study (21/40 specimens examined) has not biased the results. Pattern elements in a number of microlepidoptera have been found to surround more or fewer veins than predicted by the “alternating” and “uniform” wing-margin models, due to incomplete expression of a single pattern element (Schachat & Brown, 2015; Schachat, 2017b) or to confluence of multiple pattern elements (Schachat, 2017b; Schachat & Brown, 2018; Schachat, 2017b; Schachat, 2017a). However, the results presented here do not suggest that similar phenomena underlie the location of the CSS in *Dichromodes*.

The wing patterns of *Dichromodes* do not typically conform to any of Henke’s six categories (Fig. 4). In Henke’s categorization scheme the border symmetry system, which occurs at the distal edge of the wing, appears only when the basal symmetry system is also present. However, in *Dichromodes*, the opposite is true: pattern elements other than the CSS are more likely to appear on the wing, and to approach the complexity of a symmetry system, distal to the CSS. In *Dichromodes indicataria*, for example, the region basal to the CSS is simply mottled whereas the region distal to the CSS includes both parafocal elements and a transverse band on the wing of the male specimen (Fig. S3C), and color pattern in this area is more complex still on the wing of the female specimen (Fig. 5E).

The Geometridae whose wing patterns were included in Henke’s categorization scheme all belong to subfamilies other than Epidesmiinae. *Campaea margaritaria* Linnaeus, 1761 (Fig. 4B) belongs to the Ennominae whereas *Thera juniperata* Linnaeus, 1758 (Fig. 4C), *Eulithis testata* Linnaeus, 1761 (Fig. 4D), and *Perizoma sagittata* Fabricius, 1787 (Fig. 4E) belong to the Larentiinae—the subfamily that has been the overwhelming focus of existing studies of wing pattern in Geometridae (Henke, 1928; Süffert, 1929; Henke, 1933; Schwanwitsch, 1956; Schachat, 2019). *Dichromodes* belongs to Epidesmiinae, a lineage that diverged after Larentiinae but before Ennominae (Murillo-Ramos et al., 2019). Wing patterns in *Dichromodes* clearly suggest that Henke’s categorization scheme is unnecessarily linear and limited in scope. A more widely applicable categorization scheme for Geometridae, and perhaps for Macroheterocera as a whole, would allow the coding of the presence or absence of each element of the NGP.

Future studies should examine the relationship between wing pattern and venation in additional lineages of Macroheterocera. *Dichromodes*’ violation of all previous predictive models suggests that a focus on Geometridae will be particularly informative of the developmental diversity in macroheteroceran wing patterns. In addition to examining the relevance of the “alternating” and “uniform” wing-margin models and the location of the CSS along the costa, future studies can evaluate the co-occurrence of the various elements of the NGP in a phylogenetic context in order to inform a more useful classification scheme for macroheteroceran wing patterns that can account for homology and evolutionary history.

## Conclusions

Whereas all microlepidopteran genera that have been examined thus far have wing patterns that can be ascribed to one of the wing-margin models, *Dichromodes* is the second of two geometrid genera that have been examined in this context and both genera violate nearly all predictions based on other lineages of Lepidoptera. The location of the central symmetry system in *Dichromodes* is not constrained by venation along the costa, which suggests that venation does not influence the development of wing pattern in this genus to nearly the same extent as in certain other Macroheterocera and in butterflies. The distribution of points where the CSS terminates in *Dichromodes* is neither normal nor uniform. Because parafocal elements are confined to the termen in *Dichromodes*, the uniform wing-margin model appears to be of little or no relevance to this genus. However there is also no evidence that the alternating wing-margin model has any predictive value for *Dichromodes*. These findings raise the question of whether predictions based on other lineages of Lepidoptera are irrelevant to Geometridae and possibly a wider swath of Macroheterocera.

##  Supplemental Information

10.7717/peerj.8263/supp-1 Supplemental Information 1 Code for performing the K-S tests

10.7717/peerj.8263/supp-2 Supplemental Information 2 Wing patterns in the genus *Dichromodes*, with wing venation superimposed, continued In these specimens, the distal edge of the central symmetry system reaches the costa at Sc. Arrows indicate the point where the distal edge of the central symmetry system terminates along the costa. (A) *D. anelictis*, female. (B) *D. anelictis*, male. (C) *D. aristadelpha*, male. (D) *D. denticulata*, male. (E) *D. mesopor*, male. (F) *D. rufula*, male.

10.7717/peerj.8263/supp-3 Supplemental Information 3 Wing patterns in the genus *Dichromodes*, with wing venation superimposed, continued In these specimens, the distal edge of the central symmetry system reaches the costa between Sc and R. Arrows indicate the point where the distal edge of the central symmetry system terminates along the costa. (A) *D. ainaria*, female. (B) *D. ainaria*, male. (C) *D. compostis*, female. (D) *D. compostis*, male. (E) *D. explanata*, male. (F) *D. exsignata*, male.

10.7717/peerj.8263/supp-4 Supplemental Information 4 Wing patterns in the genus *Dichromodes*, with wing venation superimposed, continued In these specimens, the distal edge of the central symmetry system reaches the costa between Sc and R. Arrows indicate the point where the distal edge of the central symmetry system terminates along the costa. (A) *D. fulvida*, female. (B) *D. fulvida*, male. (C) *D. indicataria*, male. (D) *D. orthotis*, male.

10.7717/peerj.8263/supp-5 Supplemental Information 5 Wing patterns in the genus *Dichromodes*, with wing venation superimposed, continued In these specimens, the distal edge of the central symmetry system (CSS) reaches the costa between Sc and R. Arrows indicate the point where the distal edge of the central symmetry system terminates along the costa. (A) *D. rimosa*, male. (B) *D. rufilinea*, female. (C) *D. scothima*, female. (D) *D. triparata*, female.

10.7717/peerj.8263/supp-6 Supplemental Information 6 Wing patterns in the genus *Dichromodes*, with wing venation superimposed, continued In these specimens, the distal edge of the central symmetry system reaches the costa at R. Arrows indicate the point where the distal edge of the central symmetry system terminates along the costa. (A) *D. estigmaria*, female. (B) *D. estigmaria*, male. (C) *D. explanata*, male. (D) *D. mesotoma*, female. (E) *D. molybdaria*, female. (F) *D. molybdaria*, male.

10.7717/peerj.8263/supp-7 Supplemental Information 7 Wing patterns in the genus *Dichromodes*, with wing venation superimposed, continued In these specimens, the distal edge of the central symmetry system reaches the costa between R and Rs_1 (A) or at Rs_1 (B-D). Arrows indicate the point where the distal edge of the central symmetry system terminates along the costa. (A) *D. atrosignata*, female. (B) *D. confluaria*, male. (C) *D. leptozona*, female. (D) *D. leptozona*, male.

10.7717/peerj.8263/supp-8 Supplemental Information 8 Wing patterns in the genus *Dichromodes*, with wing venation superimposed, continued In these specimens, the distal edge of the central symmetry system reaches the costa at Rs_1. Arrows indicate the point where the distal edge of the central symmetry system terminates along the costa. (A) *D. ornata*, female. (B) *D. ornata*, male. (C) *D. stilbiata*, female. (D) *D. stilbiata*, male.

## Additional Information and Declarations

### Data Availability

The following information was supplied regarding data availability: The data is a short vector and available as a vector in a Supplemental File. All specimens examined here are in the Australian National Insect Collection: *D. molybdaria*, male: 31-036852; *D. molybdaria*, female: 31-036853; *D. mesotoma*, female: 31-036854; *D. ainaria*, male: 31-036855; D. ainaria, female: 31-036856; *D. scothima*, female: 31-036857; *D. rufula*, male: 31-036858; *D. fulvida*, male: 31-036859; *D. fulvida*, female: 31-036860; *D. compostis*, male: 31-036861; *D. compostis*, female: 31-036862; *D. orthotis*, male: 31-036863; *D. anelictis*, male: 31-036864; *D. anelictis*, female: 31-036865; *D. orthozona*, female: 31-036866; *D. deniculata*, male: 31-036867; *D. aristadelpha*, male: 31-036868; *D. paratacta*, male: 31-036869; *D. confluaria*, male: 31-036870; *D. explanata*, male: 31-036871; *D. explanata*, female: 31-036872; *D. exsignata*, female: 31-036873; *D. semicanescens*, male: 31-036874; *D. leptozona*, male: 31-036875; *D. leptozona*, female: 31-036876; *D. atrosignata*, male: 31-036877; *D. atrosignata*, female: 31-036878; *D. indicataria*, male: 31-036879; *D. indicataria*, female: 31-036880; *D. triparata*, female: 31-036881; *D. stilbiata*, male: 31-036882; *D. stilbiata*, female: 31-036883; *D. mesodonta*, female: 31-036884; *D. rimosa*, male: 31-036885; *D. mesoporphyra*, male: 31-036886; *D. ornata*, male: 31-036887; *D. ornata*, female: 31-036888; *D. rufilinea*, female: 31-036889.
