# Supplementary figures and images for: Symmetry systems on the wings of Dichromodes Guenée (Lepidoptera: Geometridae) are unconstrained by venation
Source: PeerJ. 2020 Jan 2;8:e8263. doi: 10.7717/peerj.8263 (PMC6942684; doi:10.7717/peerj.8263)

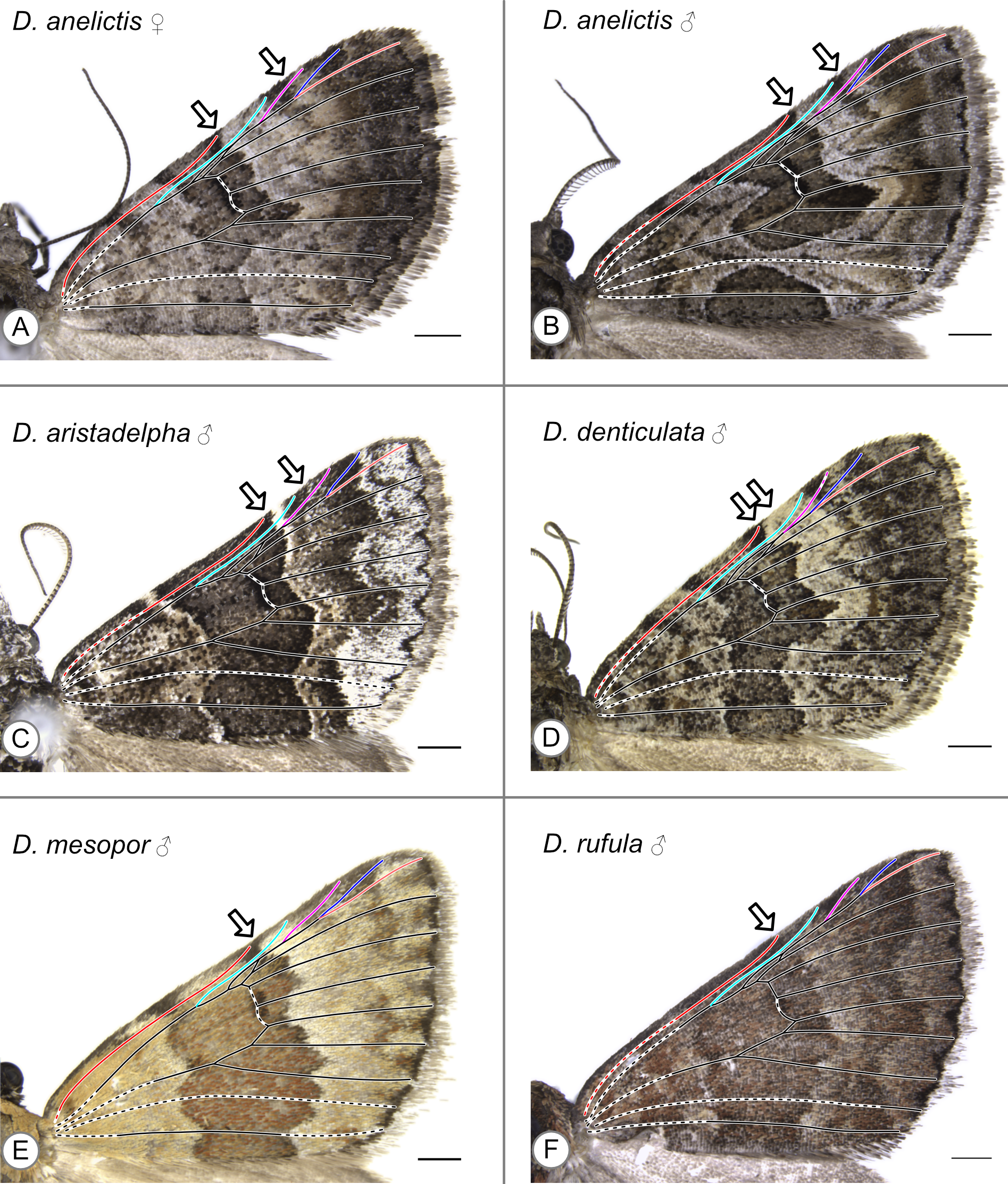

Supplement: Supplemental Information 2 — In these specimens, the distal edge of the central symmetry system reaches the costa at Sc. Arrows indicate the point where the distal edge of the central symmetry system terminates along the costa. (A) D. anelictis, female. (B) D. anelictis, male. (C) D. aristadelpha, male. (D) D. denticulata, male. (E) D. mesopor, male. (F) D. rufula, male. [file peerj-08-8263-s002.png]

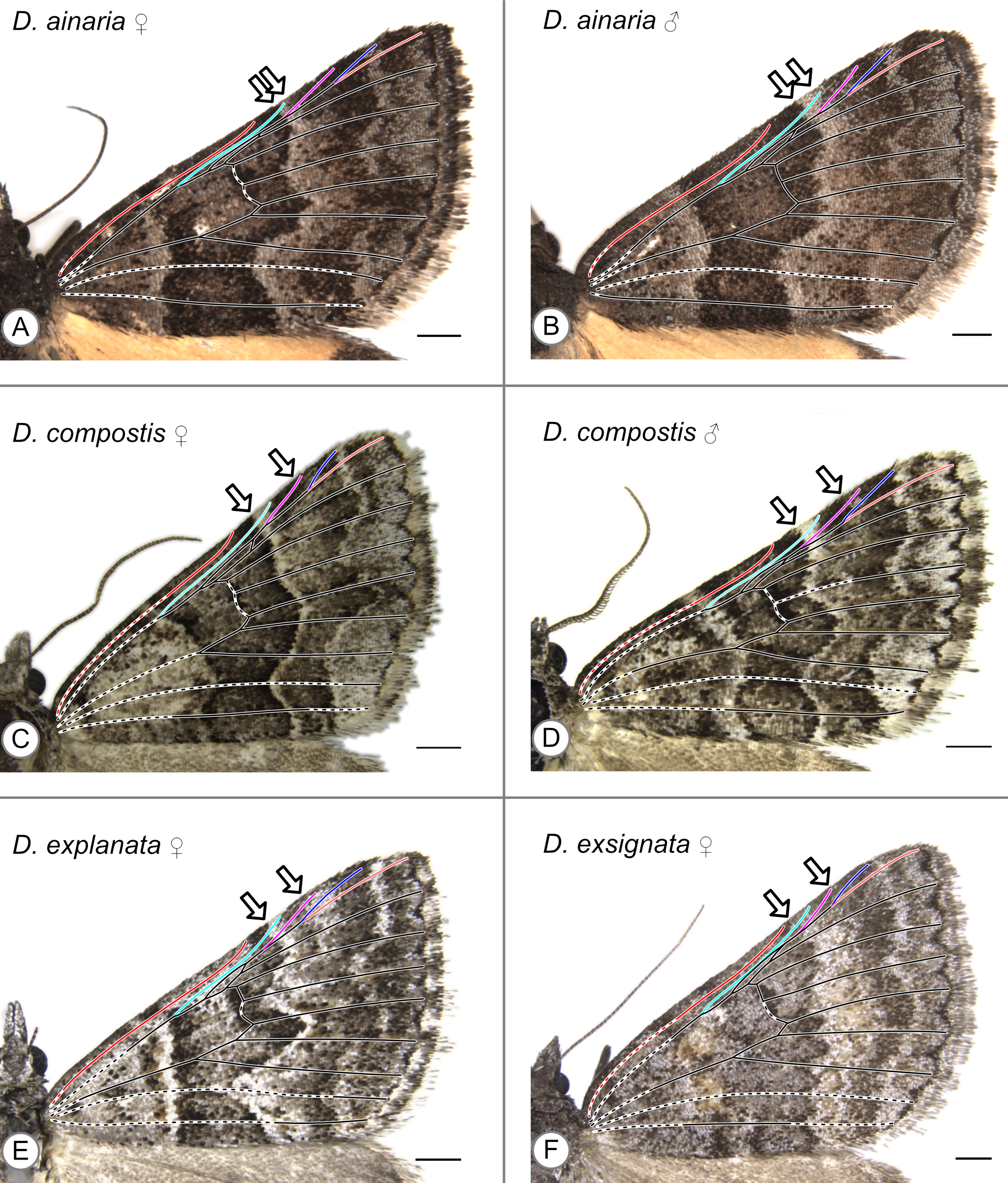

Supplement: Supplemental Information 3 — In these specimens, the distal edge of the central symmetry system reaches the costa between Sc and R. Arrows indicate the point where the distal edge of the central symmetry system terminates along the costa. (A) D. ainaria, female. (B) D. ainaria, male. (C) D. compostis, female. (D) D. compostis, male. (E) D. explanata, male. (F) D. exsignata, male. [file peerj-08-8263-s003.png]

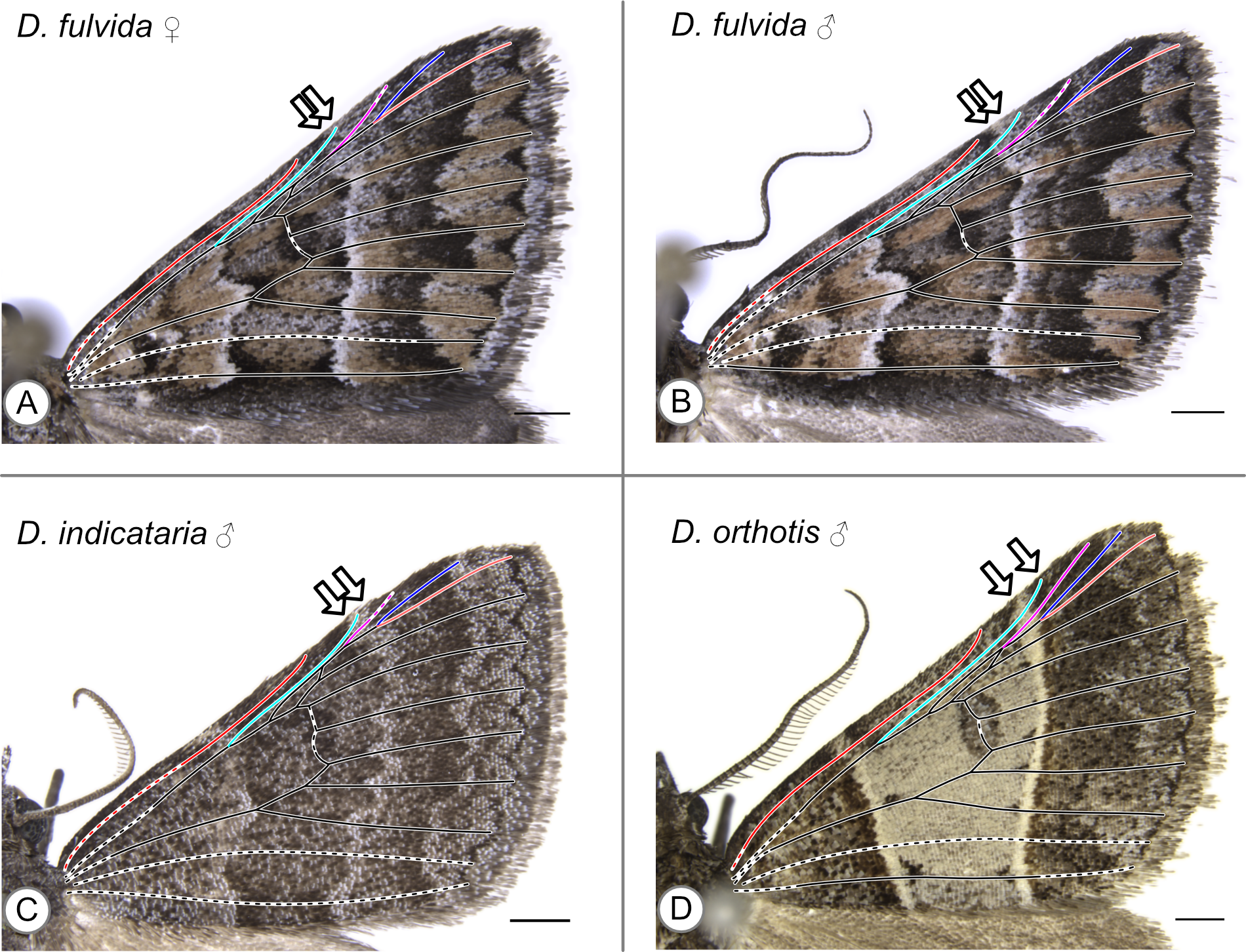

Supplement: Supplemental Information 4 — In these specimens, the distal edge of the central symmetry system reaches the costa between Sc and R. Arrows indicate the point where the distal edge of the central symmetry system terminates along the costa. (A) D. fulvida, female. (B) D. fulvida, male. (C) D. indicataria, male. (D) D. orthotis, male. [file peerj-08-8263-s004.png]

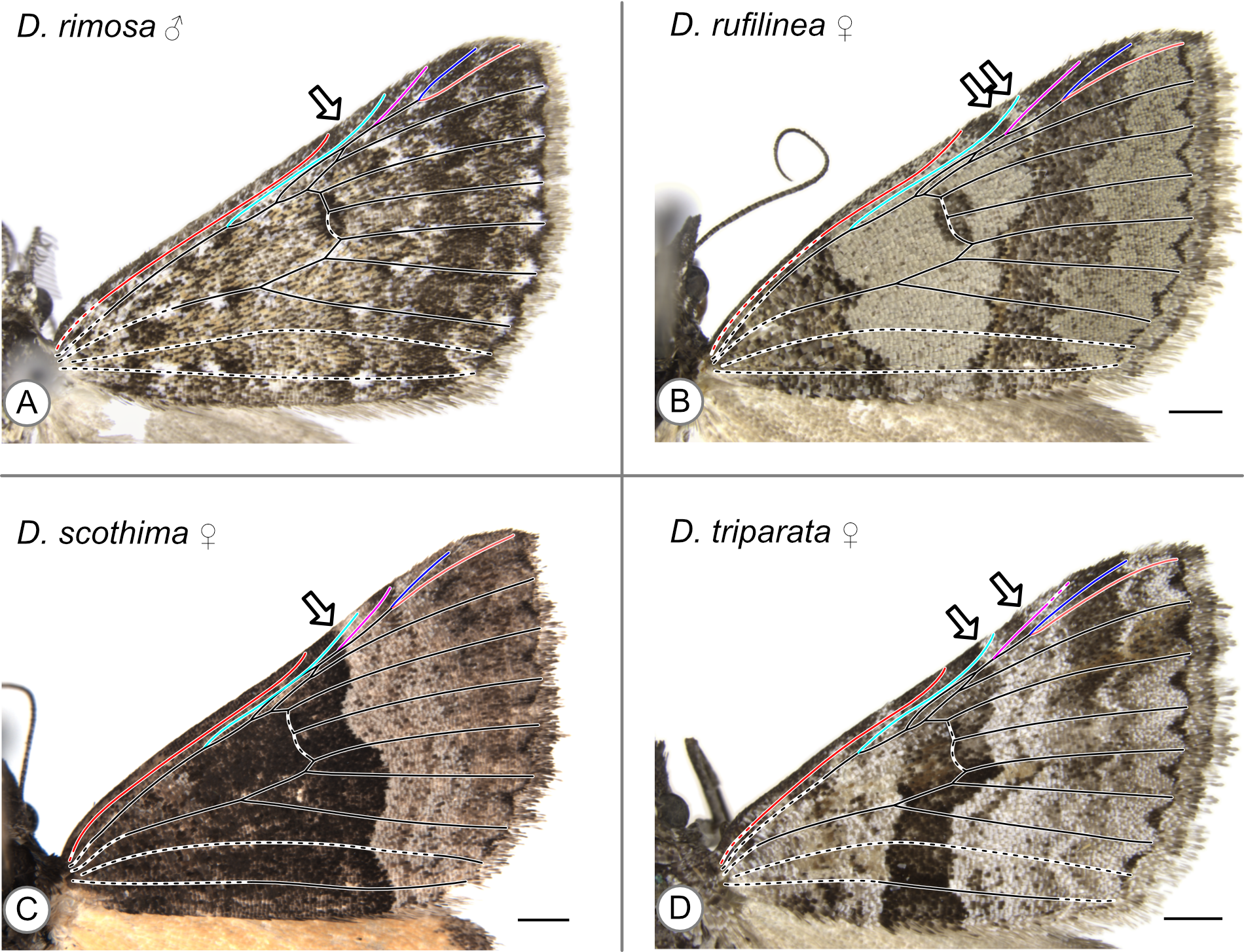

Supplement: Supplemental Information 5 — In these specimens, the distal edge of the central symmetry system (CSS) reaches the costa between Sc and R. Arrows indicate the point where the distal edge of the central symmetry system terminates along the costa. (A) D. rimosa, male. (B) D. rufilinea, female. (C) D. scothima, female. (D) D. triparata, female. [file peerj-08-8263-s005.png]

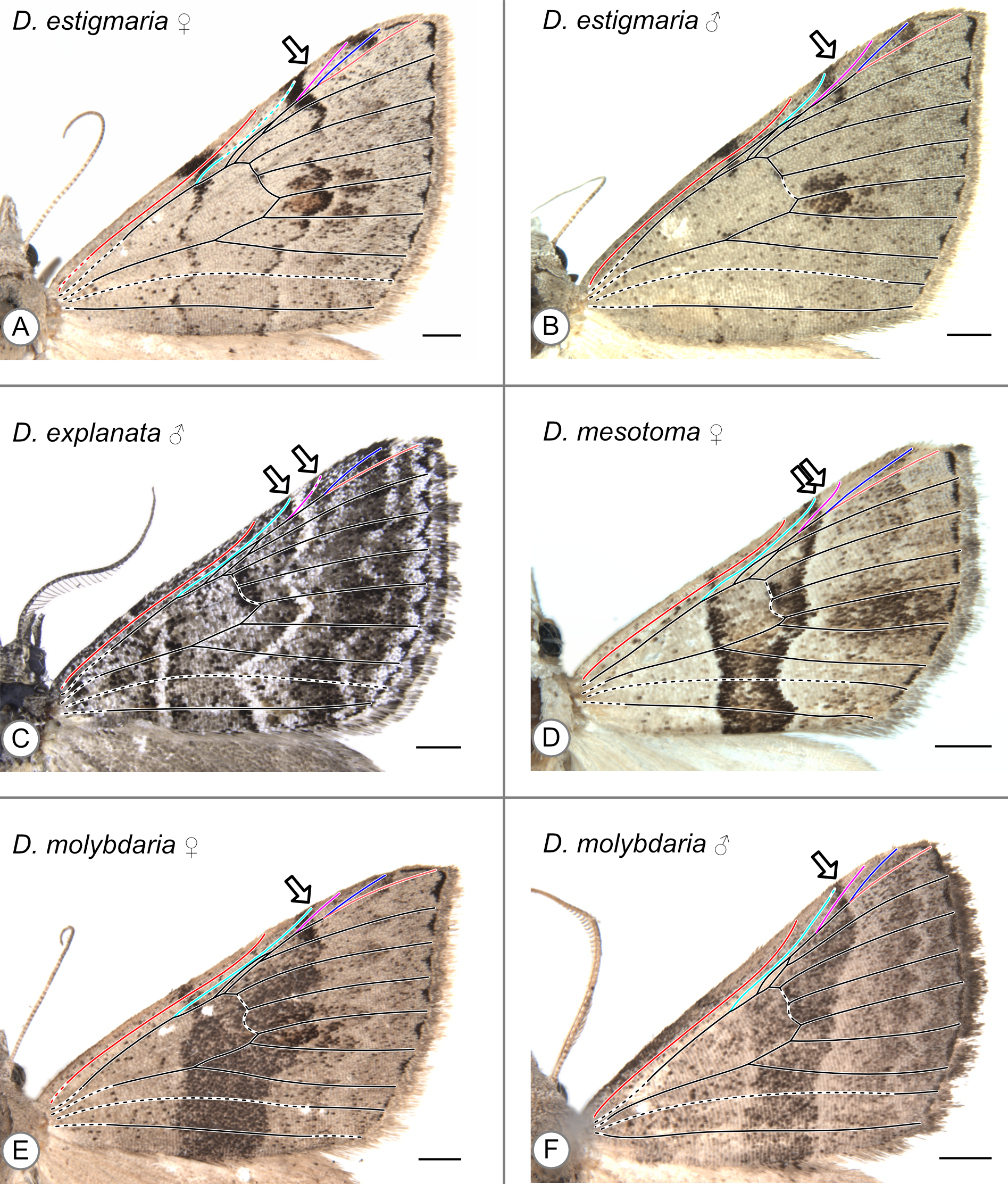

Supplement: Supplemental Information 6 — In these specimens, the distal edge of the central symmetry system reaches the costa at R. Arrows indicate the point where the distal edge of the central symmetry system terminates along the costa. (A) D. estigmaria, female. (B) D. estigmaria, male. (C) D. explanata, male. (D) D. mesotoma, female. (E) D. molybdaria, female. (F) D. molybdaria, male. [file peerj-08-8263-s006.png]

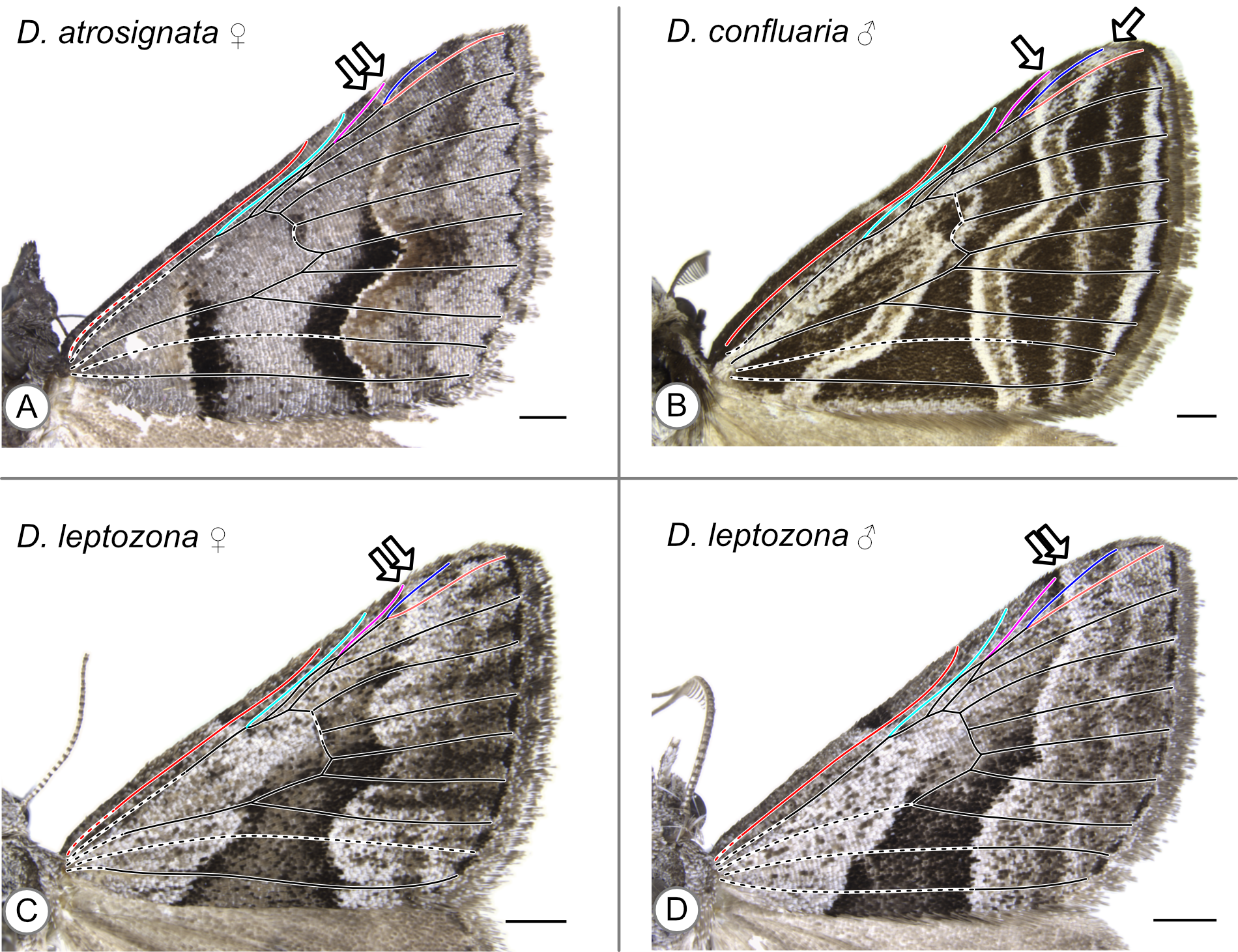

Supplement: Supplemental Information 7 — In these specimens, the distal edge of the central symmetry system reaches the costa between R and Rs_1 (A) or at Rs_1 (B-D). Arrows indicate the point where the distal edge of the central symmetry system terminates along the costa. (A) D. atrosignata, female. (B) D. confluaria, male. (C) D. leptozona, female. (D) D. leptozona, male. [file peerj-08-8263-s007.png]

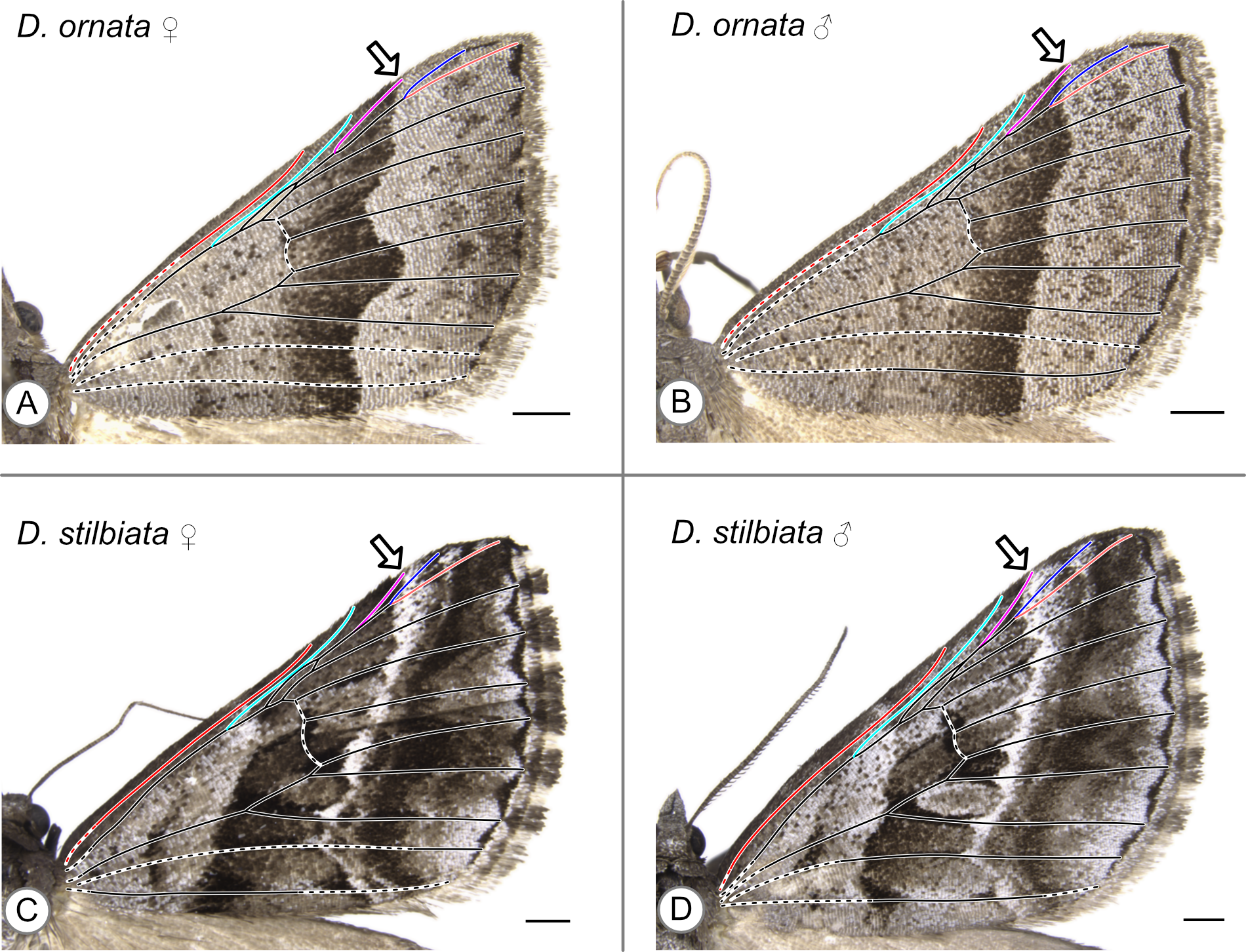

Supplement: Supplemental Information 8 — In these specimens, the distal edge of the central symmetry system reaches the costa at Rs_1. Arrows indicate the point where the distal edge of the central symmetry system terminates along the costa. (A) D. ornata, female. (B) D. ornata, male. (C) D. stilbiata, female. (D) D. stilbiata, male. [file peerj-08-8263-s008.png]
